# Challenges and Solutions for Better Management of Side Effects in Geriatric Oncology

**DOI:** 10.7759/cureus.59941

**Published:** 2024-05-09

**Authors:** Shampa Ghosh, Jitendra Kumar Sinha

**Affiliations:** 1 Biomedical Sciences, GloNeuro, Noida, IND

**Keywords:** cognition, wearable technology, yoga, mental health, telehealth, precision model of cancer therapy, fraility, geriatric oncology

## Abstract

This editorial discusses the difficulties encountered in the management of cancer among the geriatric population. Although cancer research has made substantial advancements, treatments frequently fail to consider the long-lasting consequences and adverse effects on elderly people. We advocate for enhanced geriatric oncology care, embodying enhanced evaluation techniques, the incorporation of complementary therapies, and the utilisation of wearable technologies for remote surveillance. Additionally, we suggest modifying future clinical trials to take into account the cognitive well-being of senior individuals. Implementing these modifications would greatly enhance cancer treatment for geriatric cancer patients.

## Editorial

The oldest case of cancer was documented in the Edwin Smith Papyrus, and it dates back to 3000 BC in Egypt [1]. Researchers and physicians have done a lot since then to decipher a significant portion of the complex biology of cancer. When we talk about cancer, the two most important questions that pop up are: what is the cause of it, and what are the treatment options? Often, the initial focus on treating cancer can overshadow the long-term effects of side effects and chronic health problems associated with it. Side effects are a major concern for everyone undergoing cancer treatment and remain a significant, persistent, and unavoidable challenge with the cancer therapies available to date. Recent studies have focused on the urgent needs of elderly cancer patients [2]. A recent analysis found that the geriatric population is not included in most of the clinical trials [3], citing many reasons. On the other hand, age is known to be one of the most significant risk factors for acquiring cancer. Accessing cancer care for the elderly is a big challenge, which worsened during the COVID-19 pandemic [4]. Limited mobility due to frailty and weak bone health poses serious challenges for elderly patients. Adding to their troubles, the side effects of cancer medications are found to be more prominent in this population. Therefore, there is an urgent need to implement changes and improvements in the treatment paradigm for geriatric oncology.

Firstly, improved geriatric oncological assessment is required, considering factors such as comorbidities, physical and cognitive functioning, frailty, and medication use. This would help in grouping the patients and devising a better precision model of cancer therapy. These assessments can be tailored for telehealth options as well, giving more leverage to the overall treatment of elderly cancer patients. Secondly, the integration of complementary therapies like yoga and meditation has been shown to significantly benefit the elderly population in reducing both cancer-related physical and mental fatigue as well as global side-effect burden [5]. Yoga substantially helps in improving cognitive flexibility and reducing persistent stress levels in older adults. Thirdly, it is highly recommended to use wearable technology to facilitate remote geriatric evaluations, thereby decreasing the need for regular in-person assessments, especially in rural and underserved areas. Finally, it would be great if we could implement adaptive clinical trial designs with a dynamic cognitive function evaluation component targeting elderly brain health. The reason is that traditional clinical trials focus primarily on biomarkers or disease progression, which completely overlooks cognitive changes in elderly participants who are at risk of neurodegenerative disorders. We believe that these steps would bring considerable positive change in the area of cancer care for the elderly population.
